# Supplementary figures and images for: Myosin light chain 3 serves as a receptor for nervous necrosis virus entry into host cells via the macropinocytosis pathway
Source: eLife. 2025 Jun 25;13:RP104772. doi: 10.7554/eLife.104772 (PMC12194134; doi:10.7554/eLife.104772)

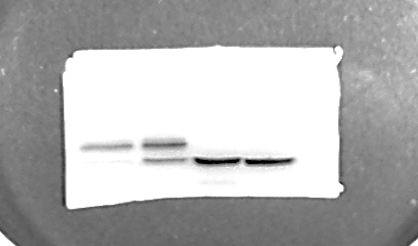

Supplement: Figure 1—source data 1. [file elife-104772-fig1-data1.zip › Figure 1-source data 1/Figure 1A-1.tif]

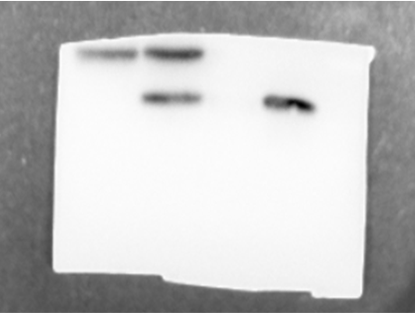

Supplement: Figure 1—source data 1. [file elife-104772-fig1-data1.zip › Figure 1-source data 1/Figure 1A-2.tif]

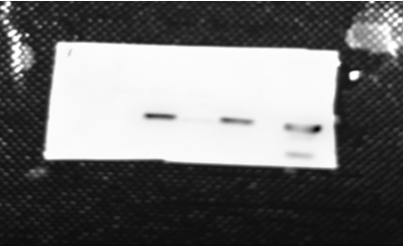

Supplement: Figure 1—source data 4. [file elife-104772-fig1-data4.zip › Figure 1-source data 4/Figure 1C-1.tif]

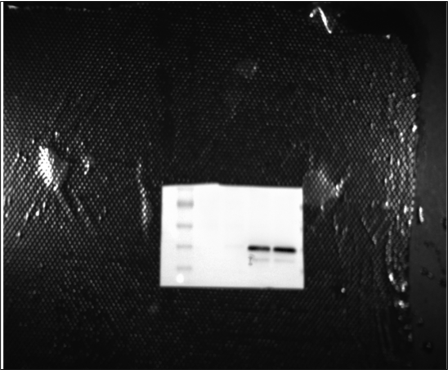

Supplement: Figure 1—source data 4. [file elife-104772-fig1-data4.zip › Figure 1-source data 4/Figure 1C-2.tif]

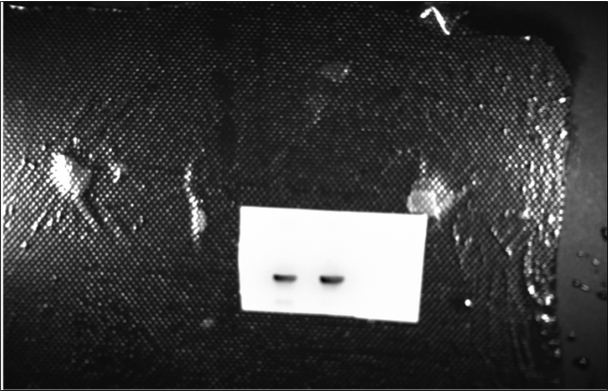

Supplement: Figure 1—source data 4. [file elife-104772-fig1-data4.zip › Figure 1-source data 4/Figure 1C-3.tif]

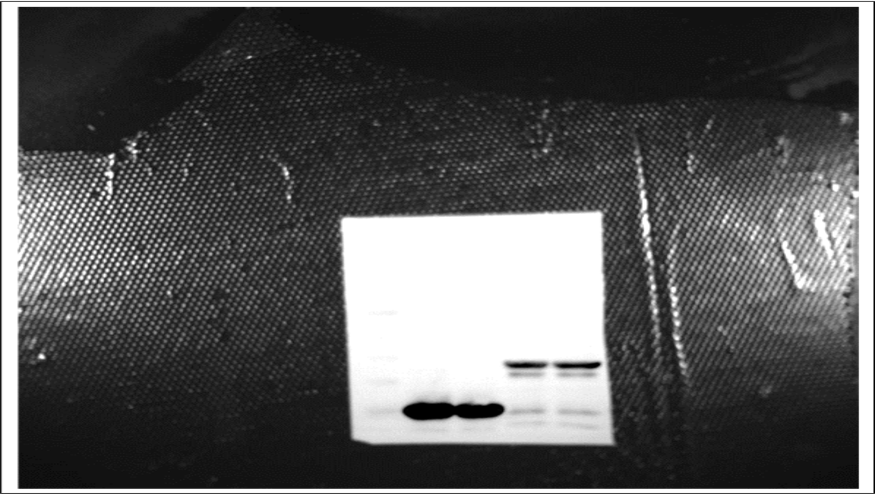

Supplement: Figure 1—source data 4. [file elife-104772-fig1-data4.zip › Figure 1-source data 4/Figure 1C-4.tif]

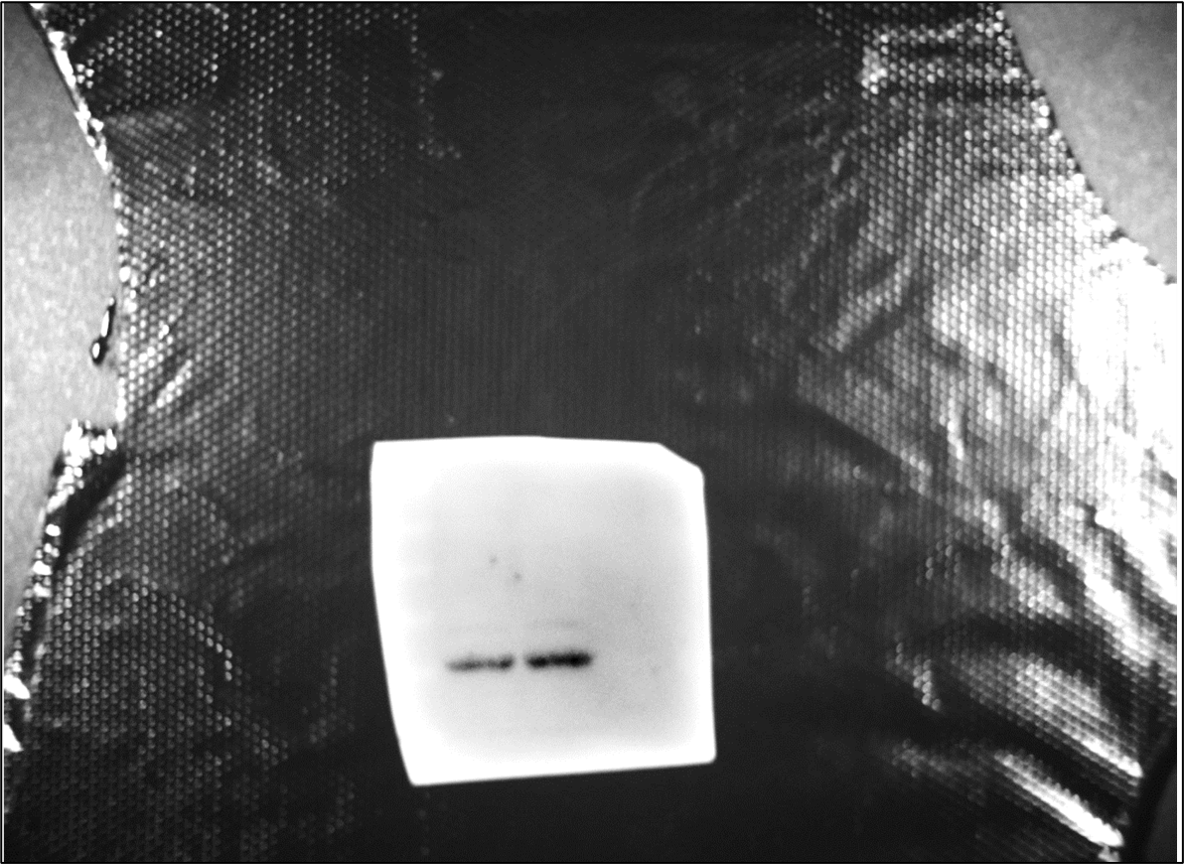

Supplement: Figure 1—source data 4. [file elife-104772-fig1-data4.zip › Figure 1-source data 4/Figure 1C-5.tif]

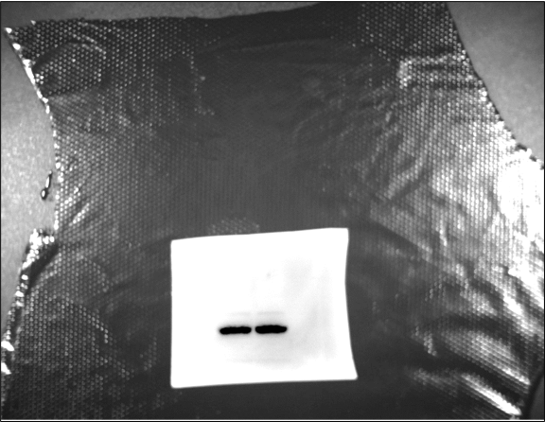

Supplement: Figure 1—source data 4. [file elife-104772-fig1-data4.zip › Figure 1-source data 4/Figure 1C-6.tif]

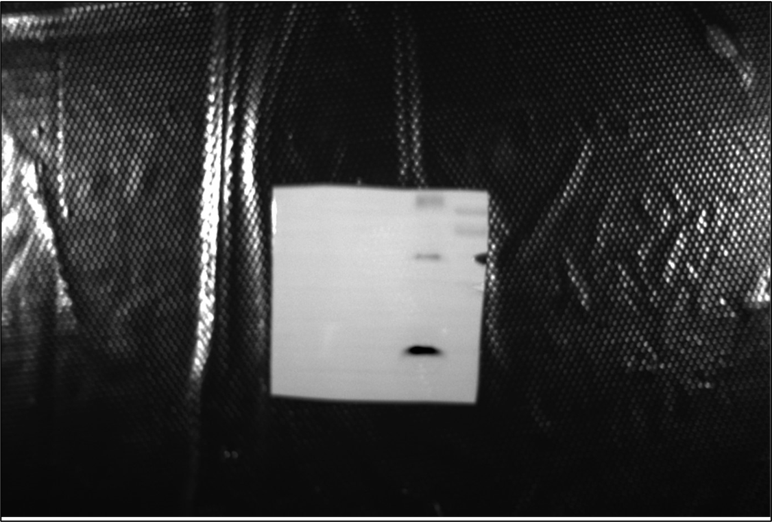

Supplement: Figure 1—source data 6. [file elife-104772-fig1-data6.zip › Figure 1-source data 6/Figure 1D-1.tif]

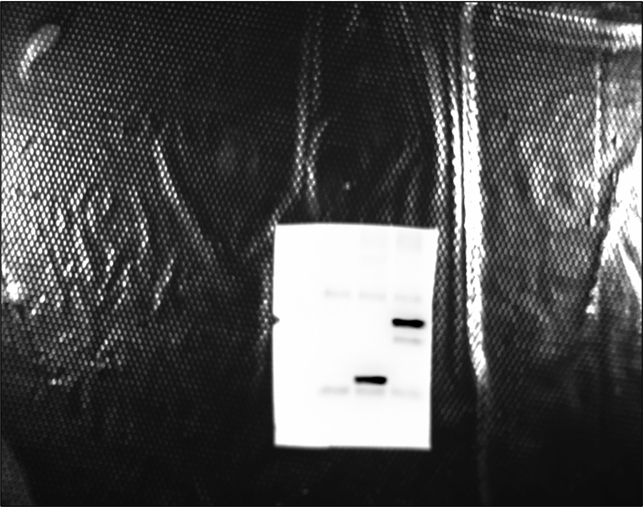

Supplement: Figure 1—source data 6. [file elife-104772-fig1-data6.zip › Figure 1-source data 6/Figure 1D-2.tif]

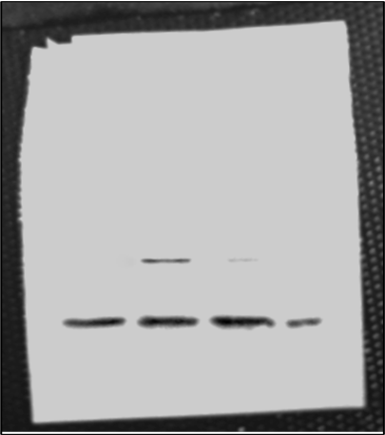

Supplement: Figure 1—source data 9. [file elife-104772-fig1-data9.zip › Figure 1-source data 9/Figure 1F-1.tif]

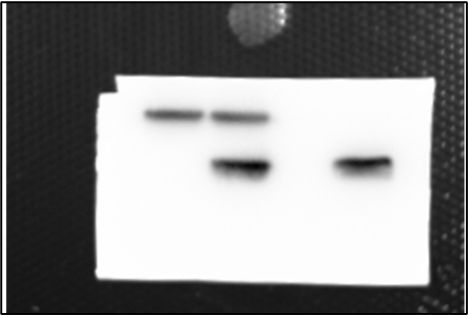

Supplement: Figure 1—source data 9. [file elife-104772-fig1-data9.zip › Figure 1-source data 9/Figure 1F-2.tif]

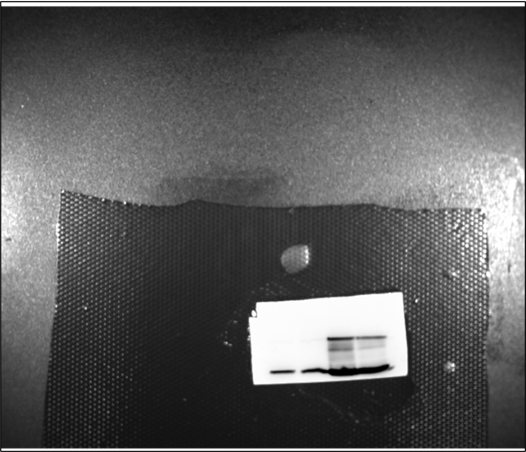

Supplement: Figure 1—source data 9. [file elife-104772-fig1-data9.zip › Figure 1-source data 9/Figure 1F-3.tif]

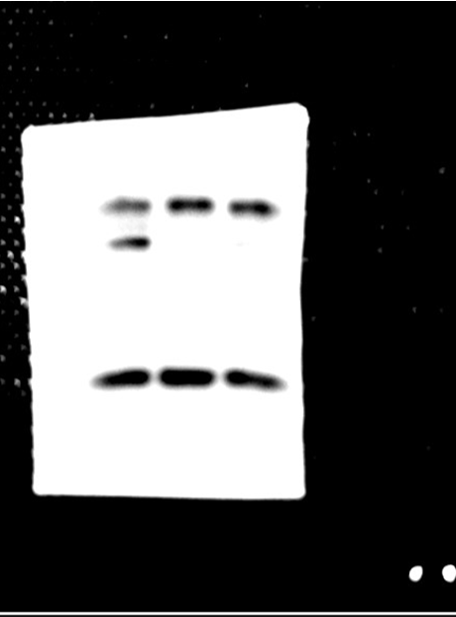

Supplement: Figure 1—source data 11. [file elife-104772-fig1-data11.zip › Figure 1-source data 11/Figure 1I-1.tif]

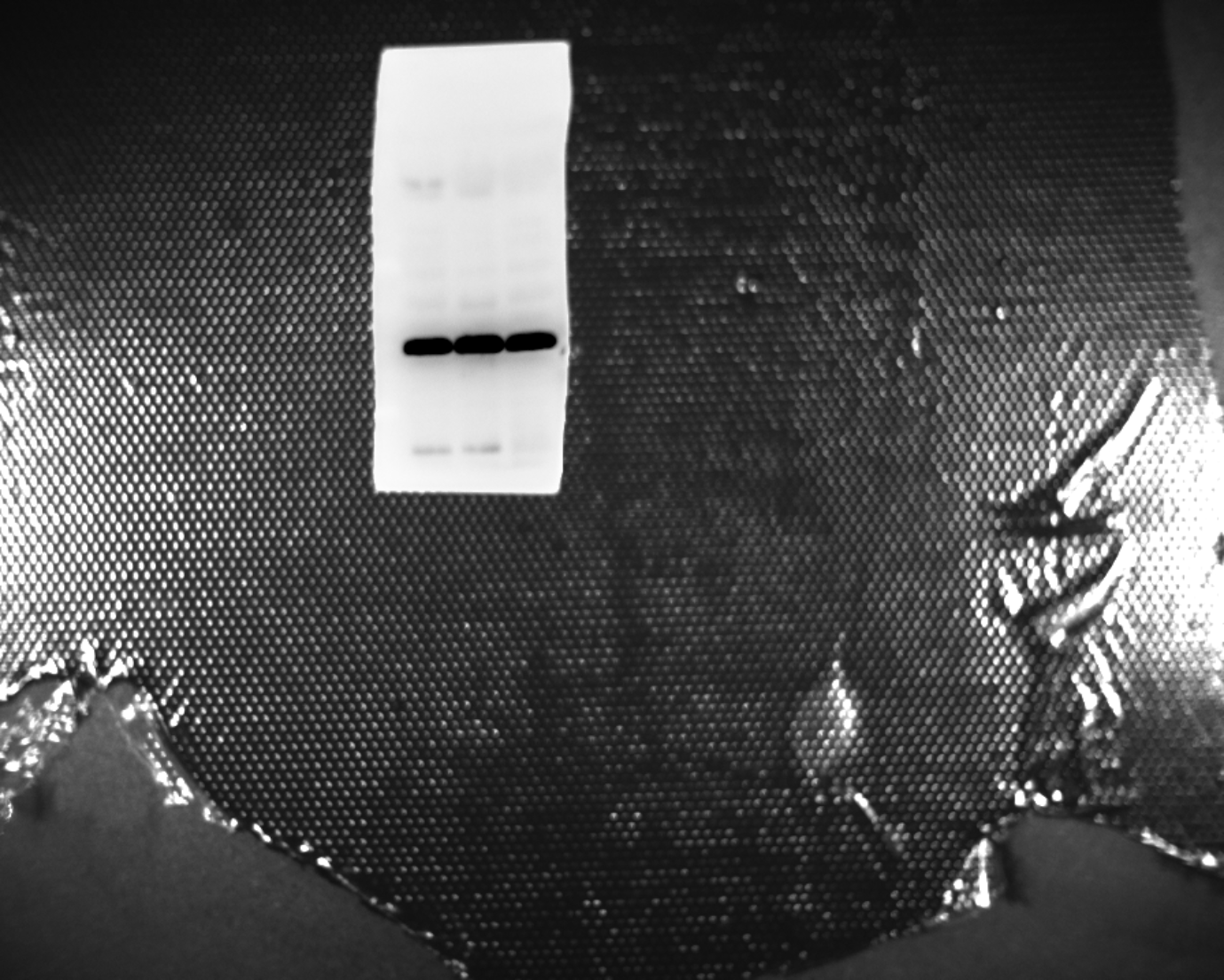

Supplement: Figure 1—source data 11. [file elife-104772-fig1-data11.zip › Figure 1-source data 11/Figure 1I-2.tif]

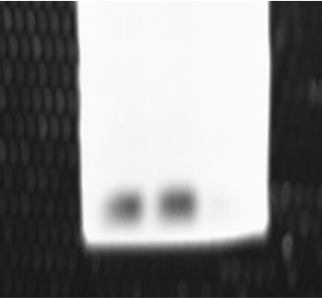

Supplement: Figure 1—source data 11. [file elife-104772-fig1-data11.zip › Figure 1-source data 11/Figure 1I-3.tif]

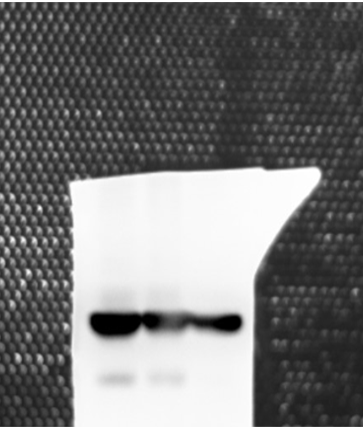

Supplement: Figure 1—source data 11. [file elife-104772-fig1-data11.zip › Figure 1-source data 11/Figure 1I-4.tif]

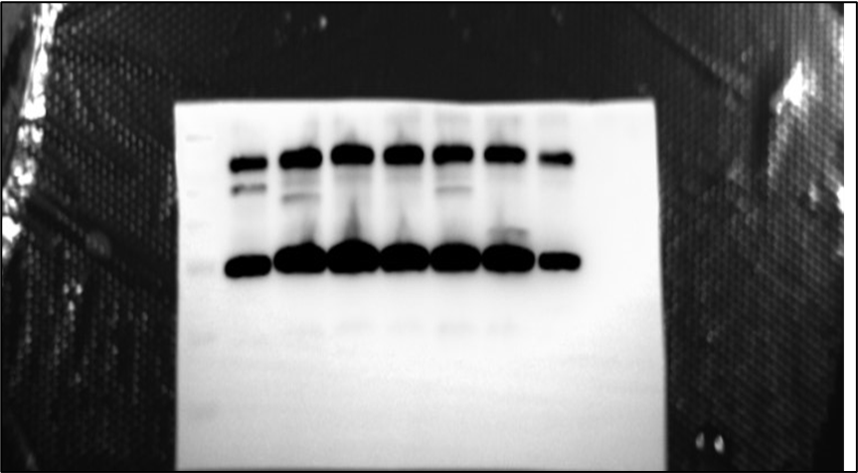

Supplement: Figure 1—source data 13. [file elife-104772-fig1-data13.zip › Figure 1-source data 13/Figure 1J-1.tif]

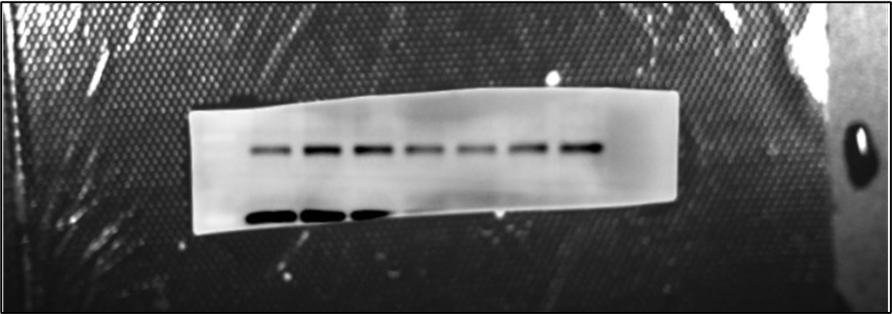

Supplement: Figure 1—source data 13. [file elife-104772-fig1-data13.zip › Figure 1-source data 13/Figure 1J-2.tif]

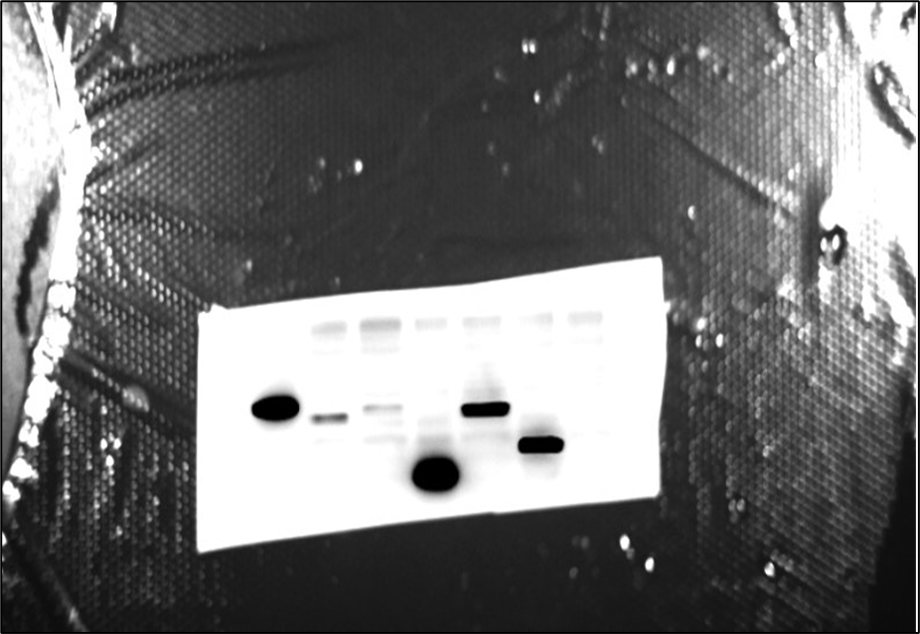

Supplement: Figure 1—source data 13. [file elife-104772-fig1-data13.zip › Figure 1-source data 13/Figure 1J-3.tif]

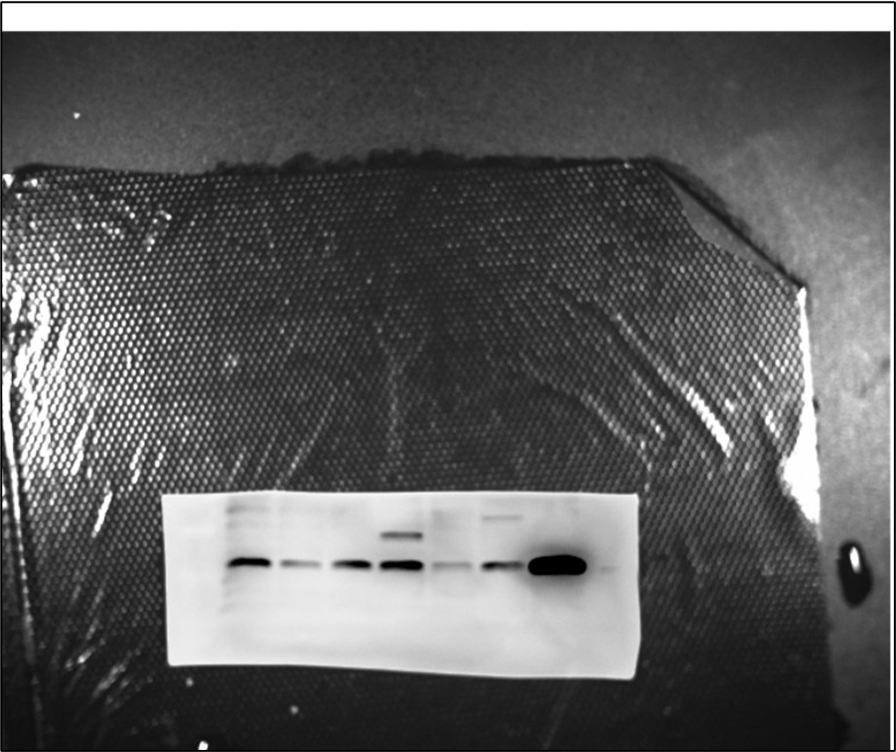

Supplement: Figure 1—source data 13. [file elife-104772-fig1-data13.zip › Figure 1-source data 13/Figure 1J-4.tif]

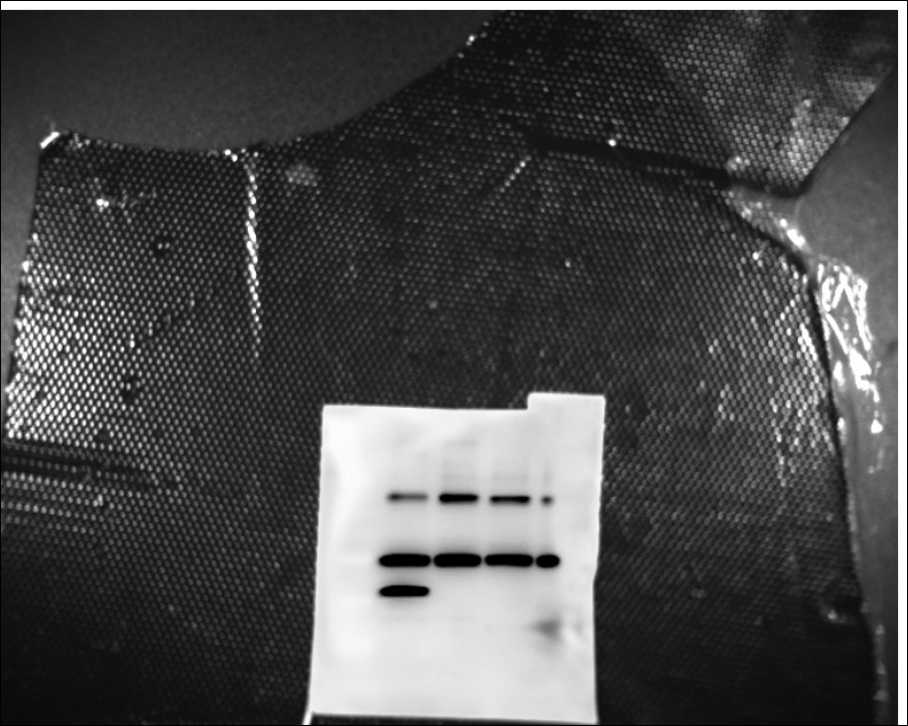

Supplement: Figure 1—figure supplement 1—source data 1. [file elife-104772-fig1-figsupp1-data1.zip › Figure 1-supplement 1-source data 1/Figure 1-supplement 1-1.tif]

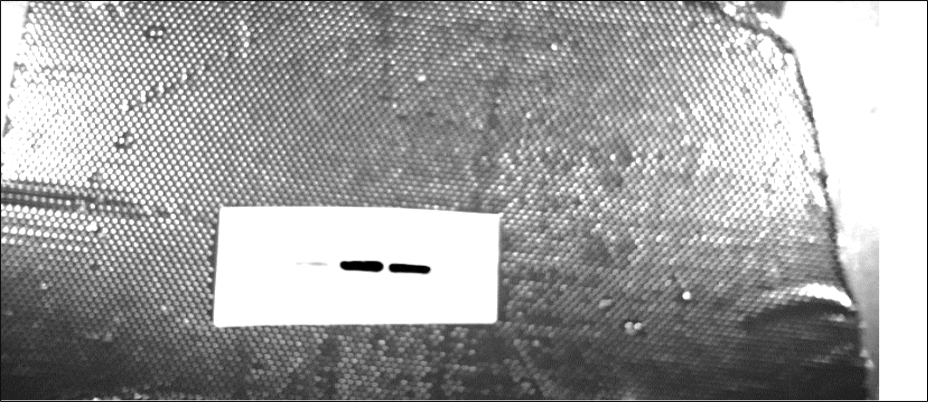

Supplement: Figure 1—figure supplement 1—source data 1. [file elife-104772-fig1-figsupp1-data1.zip › Figure 1-supplement 1-source data 1/Figure 1-supplement 1-2.tif]

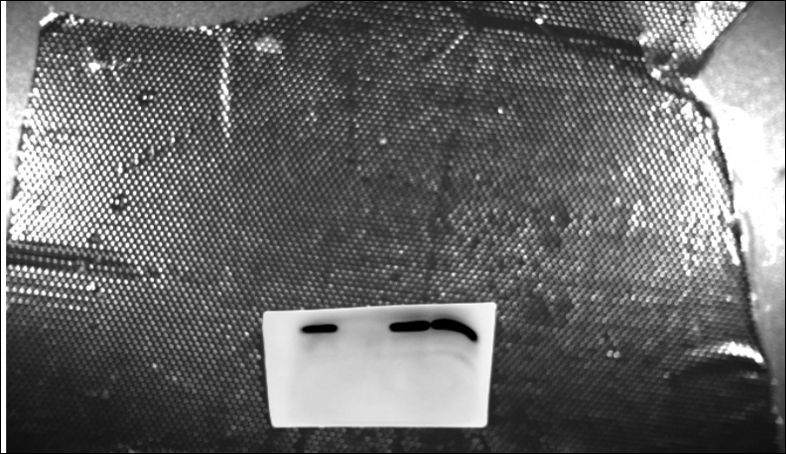

Supplement: Figure 1—figure supplement 1—source data 1. [file elife-104772-fig1-figsupp1-data1.zip › Figure 1-supplement 1-source data 1/Figure 1-supplement 1-3.tif]

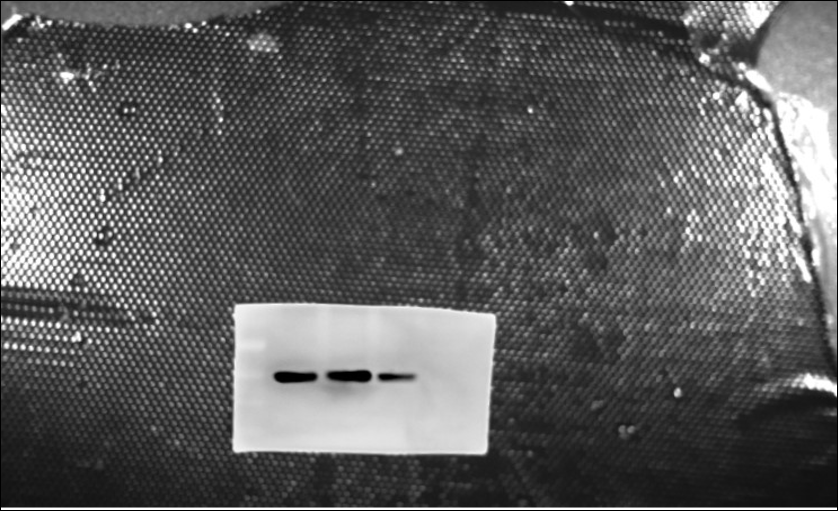

Supplement: Figure 1—figure supplement 1—source data 1. [file elife-104772-fig1-figsupp1-data1.zip › Figure 1-supplement 1-source data 1/Figure 1-supplement 1-4.tif]

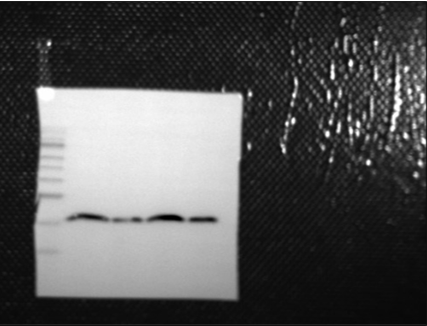

Supplement: Figure 2—source data 1. [file elife-104772-fig2-data1.zip › Figure 2-source data 1/Figure 2C.tif]

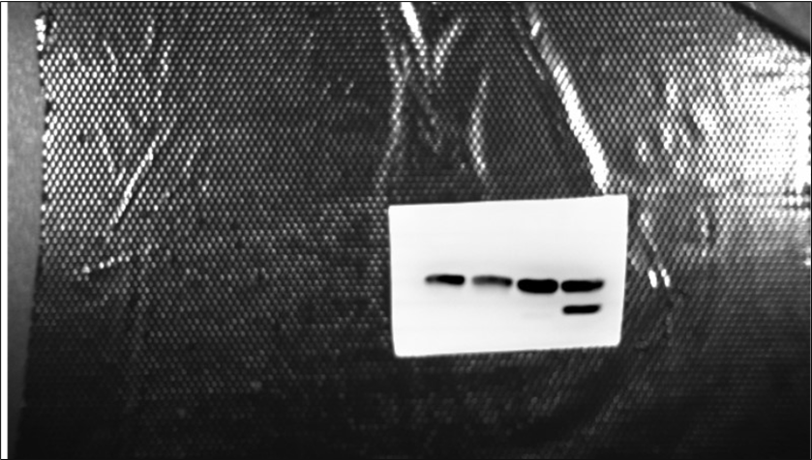

Supplement: Figure 2—source data 1. [file elife-104772-fig2-data1.zip › Figure 2-source data 1/Figure 2D.tif]

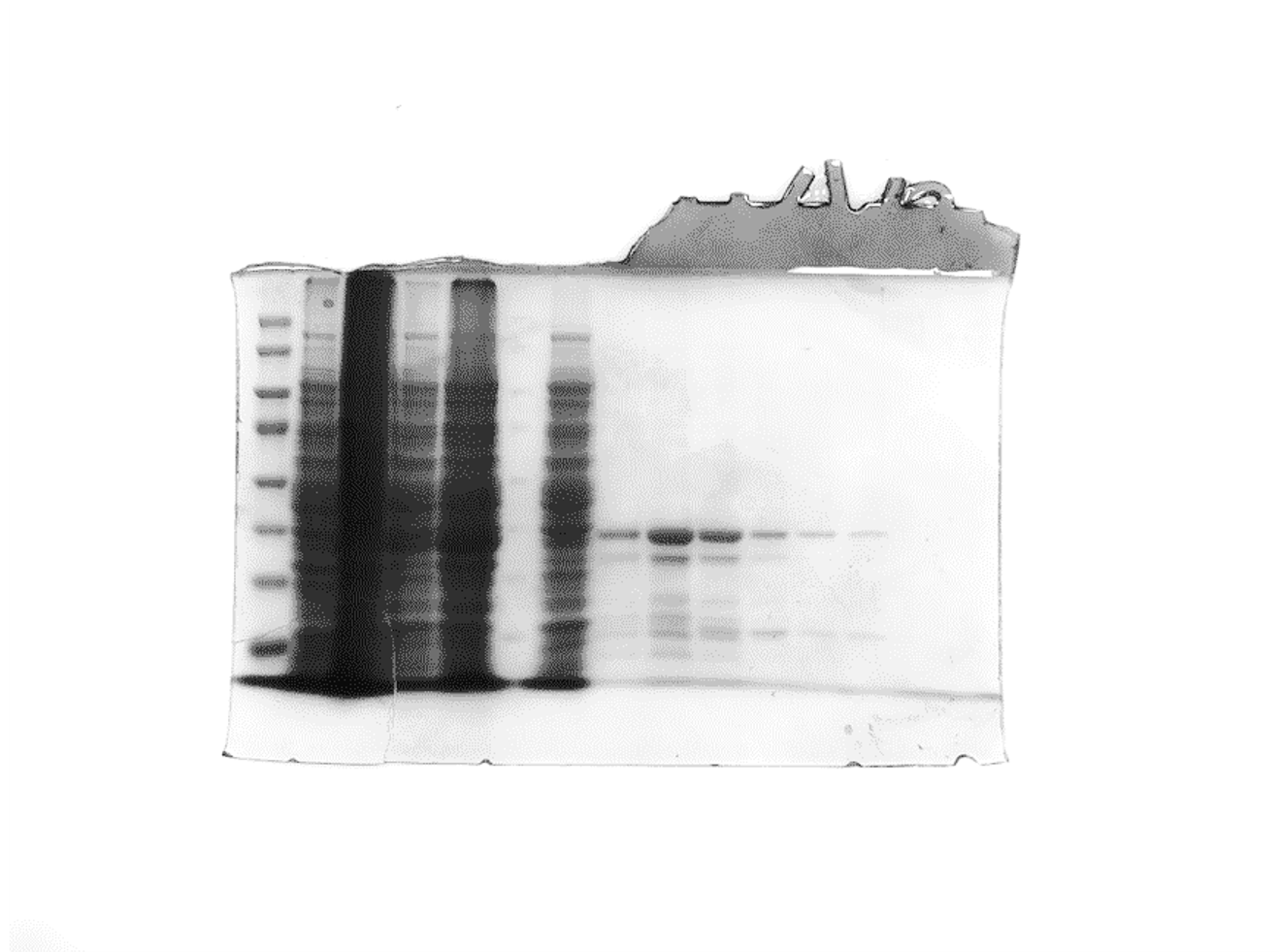

Supplement: Figure 3—figure supplement 1—source data 1. [file elife-104772-fig3-figsupp1-data1.zip › Figure 3-supplement 1-source data 1/Figure 3-supplement 1B-1.tif]

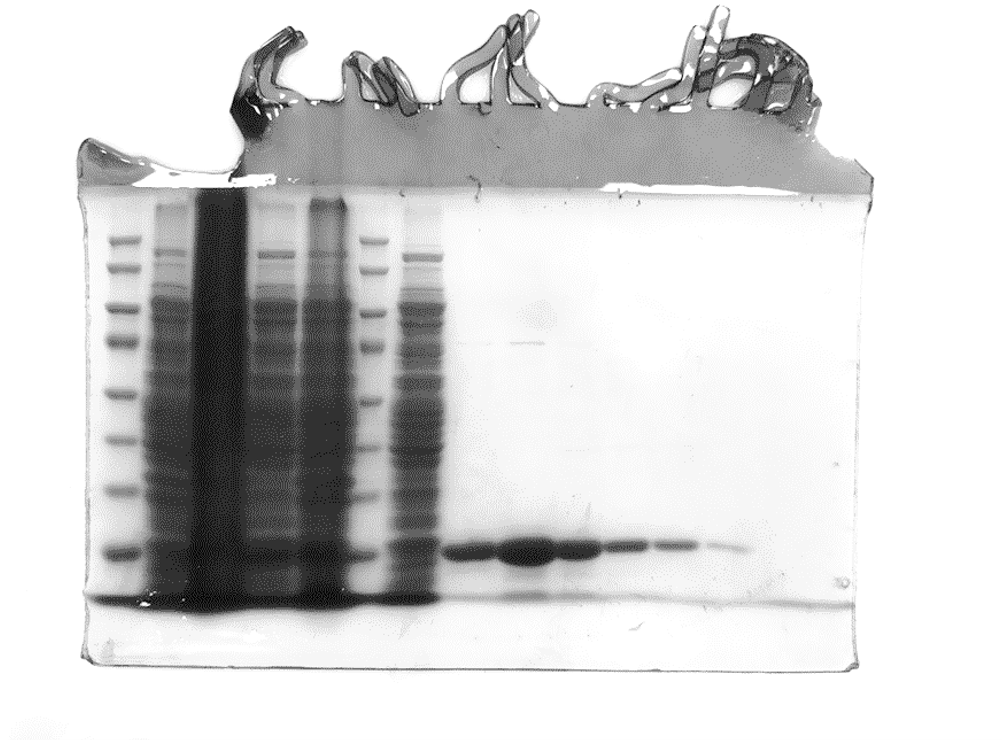

Supplement: Figure 3—figure supplement 1—source data 1. [file elife-104772-fig3-figsupp1-data1.zip › Figure 3-supplement 1-source data 1/Figure 3-supplement 1B-2.tif]

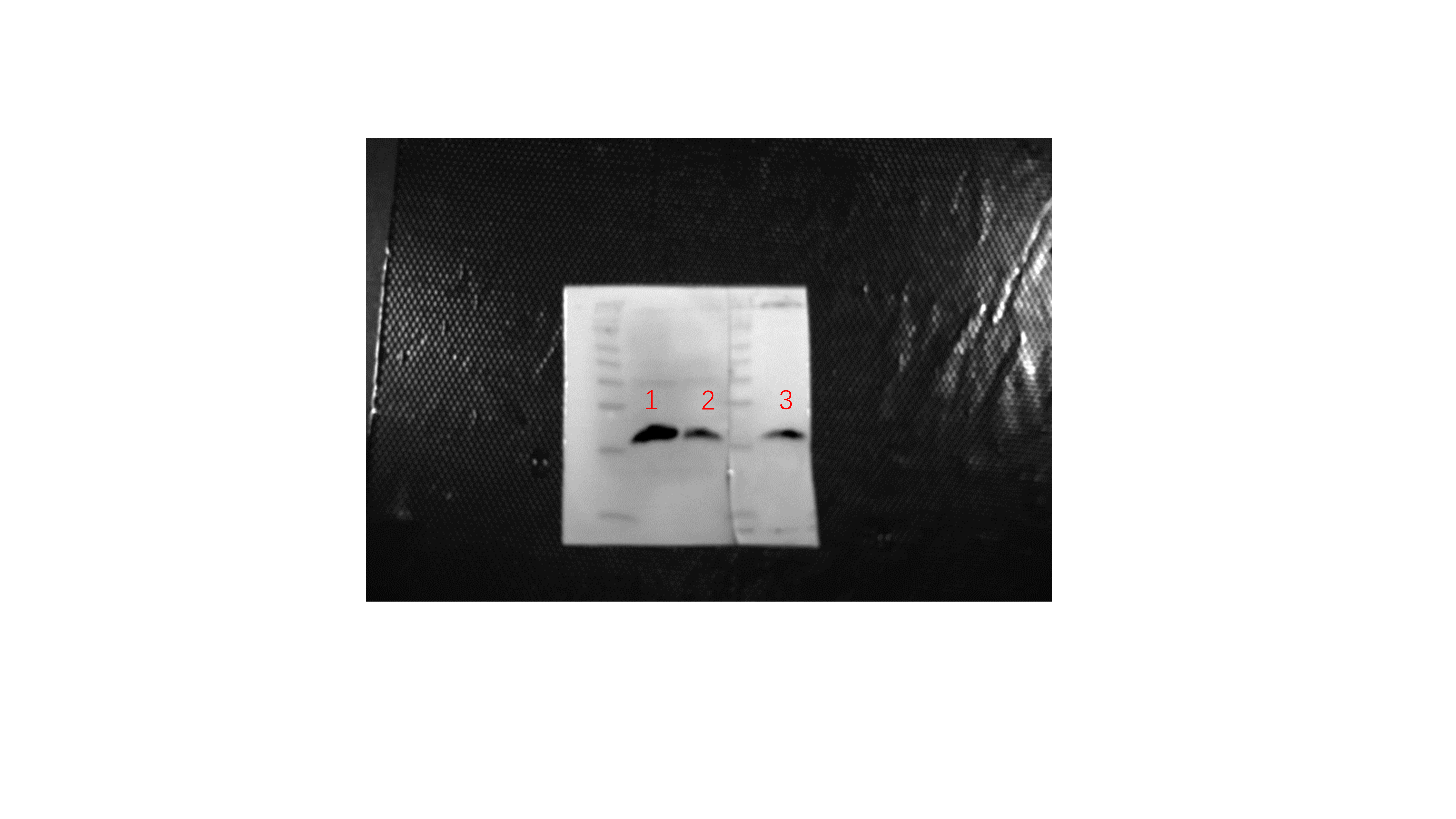

Supplement: Figure 3—figure supplement 1—source data 3. [file elife-104772-fig3-figsupp1-data3.zip › Figure 3-supplement 1-source data 3/Figure 3-supplement 1C.tif]

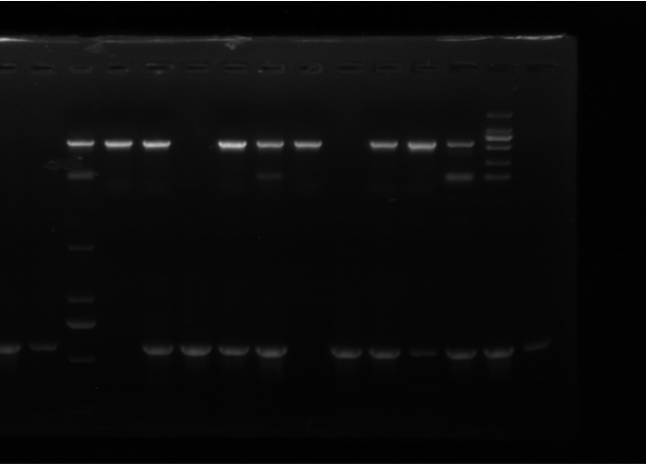

Supplement: Figure 4—source data 1. [file elife-104772-fig4-data1.zip › Figure 4-source data 1/Figure 4A-1.tif]

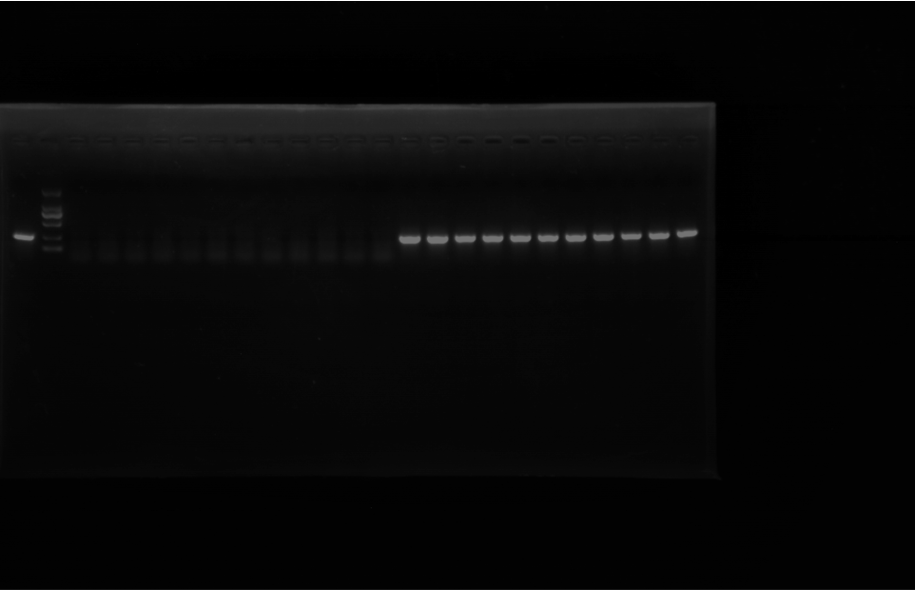

Supplement: Figure 4—source data 1. [file elife-104772-fig4-data1.zip › Figure 4-source data 1/Figure 4A-2.tif]

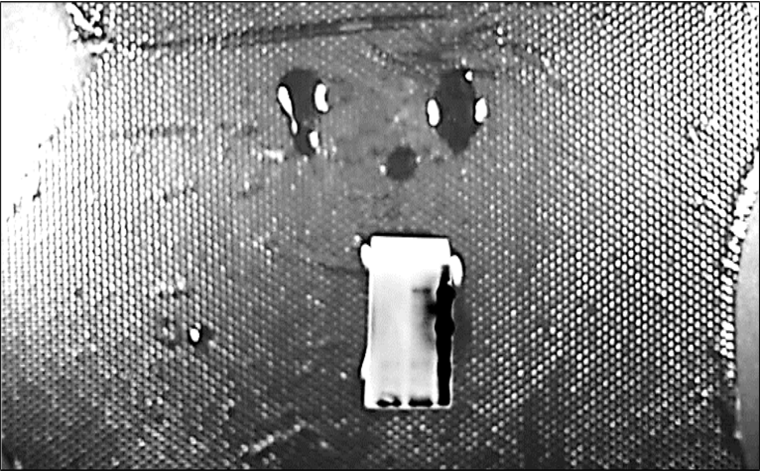

Supplement: Figure 6—source data 3. [file elife-104772-fig6-data3.zip › Figure 6-source data 3/Figure 6C-1.tif]

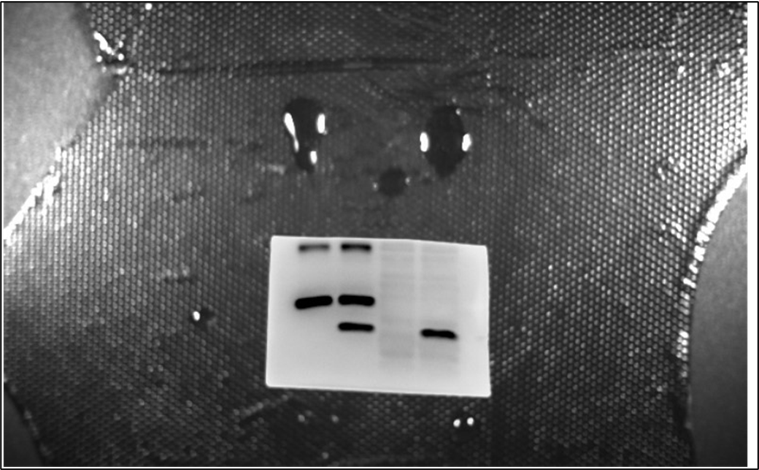

Supplement: Figure 6—source data 3. [file elife-104772-fig6-data3.zip › Figure 6-source data 3/Figure 6C-2.tif]

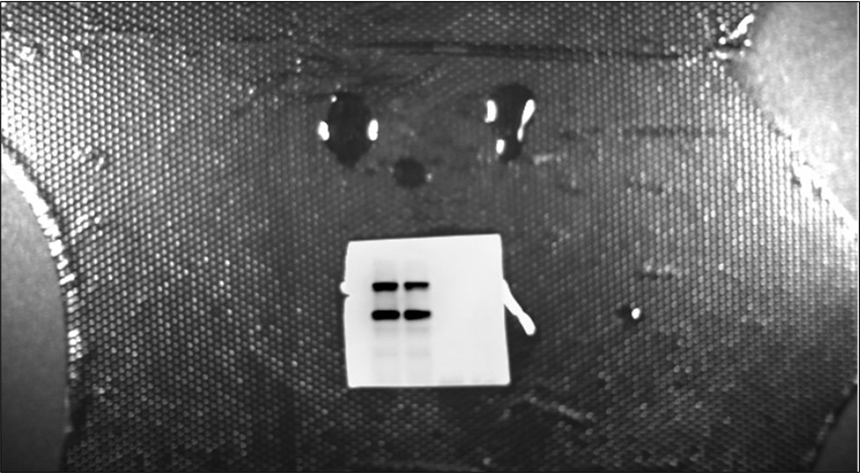

Supplement: Figure 6—source data 3. [file elife-104772-fig6-data3.zip › Figure 6-source data 3/Figure 6C-3.tif]

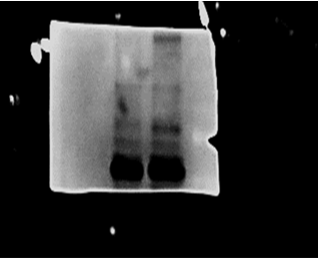

Supplement: Figure 6—source data 5. [file elife-104772-fig6-data5.zip › Figure 6-source data 5/Figure 6D-1.tif]

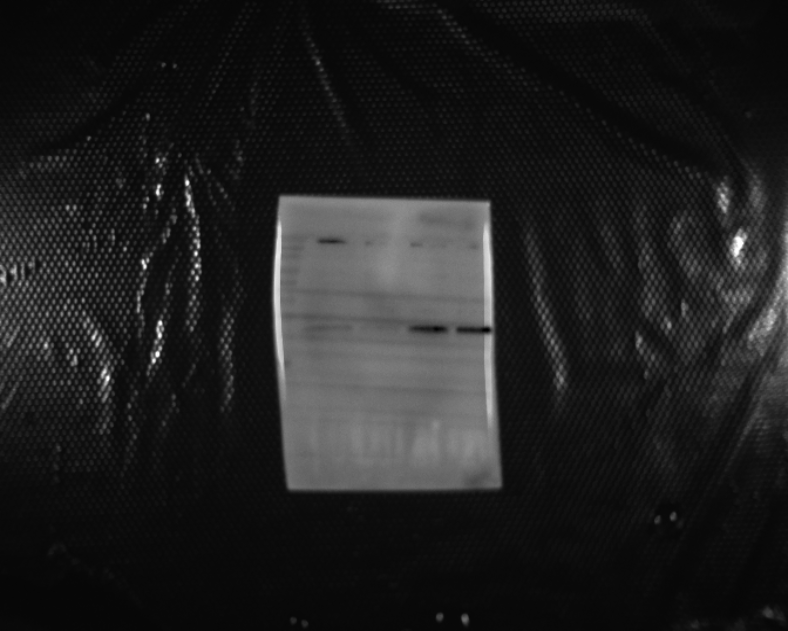

Supplement: Figure 6—source data 5. [file elife-104772-fig6-data5.zip › Figure 6-source data 5/Figure 6D-2.tif]

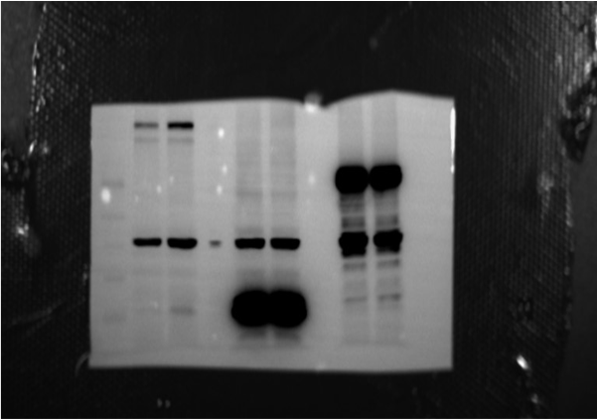

Supplement: Figure 6—source data 5. [file elife-104772-fig6-data5.zip › Figure 6-source data 5/Figure 6D-3.tif]

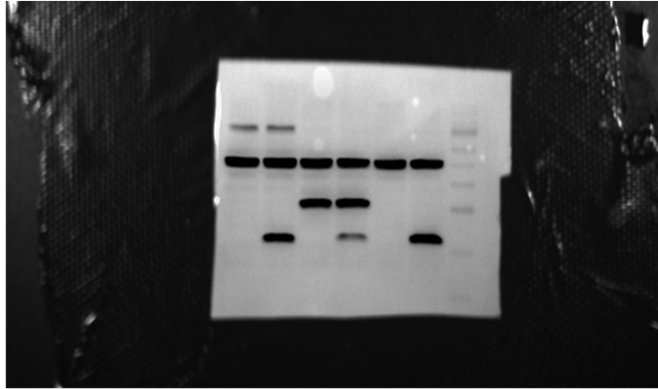

Supplement: Figure 6—source data 5. [file elife-104772-fig6-data5.zip › Figure 6-source data 5/Figure 6D-4.tif]

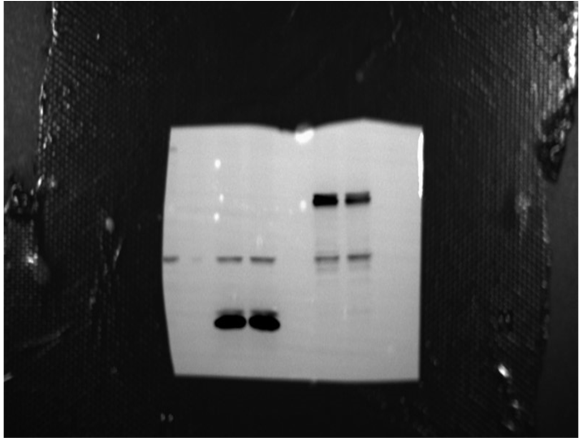

Supplement: Figure 6—source data 5. [file elife-104772-fig6-data5.zip › Figure 6-source data 5/Figure 6D-5.tif]

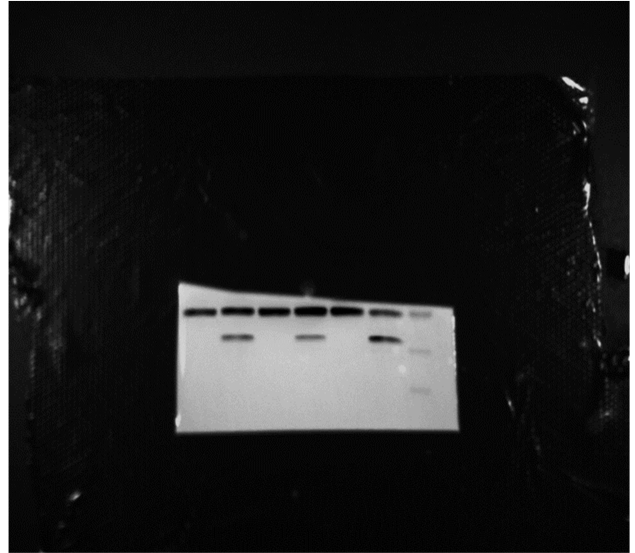

Supplement: Figure 6—source data 5. [file elife-104772-fig6-data5.zip › Figure 6-source data 5/Figure 6D-6.tif]

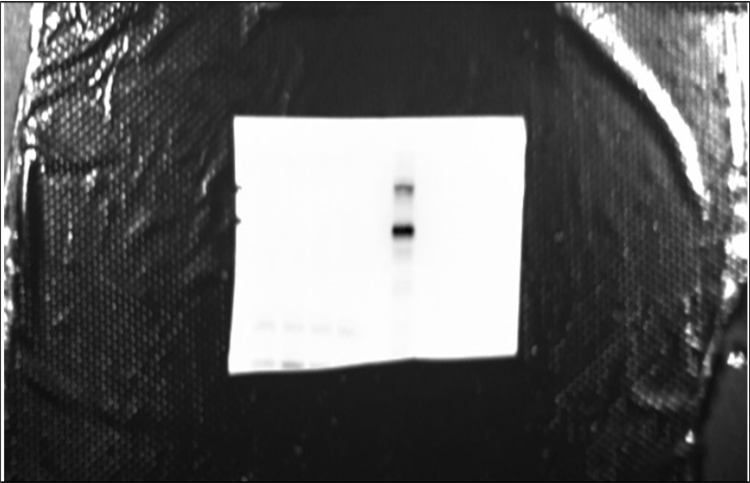

Supplement: Figure 6—source data 7. [file elife-104772-fig6-data7.zip › Figure 6-source data 7/Figure 6E-1.tif]

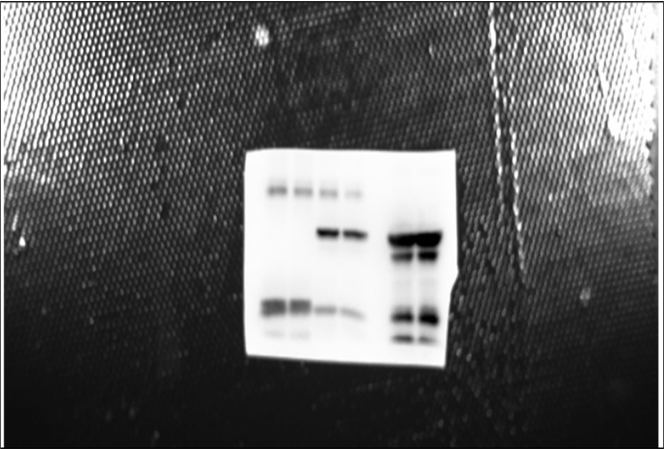

Supplement: Figure 6—source data 7. [file elife-104772-fig6-data7.zip › Figure 6-source data 7/Figure 6E-2.tif]

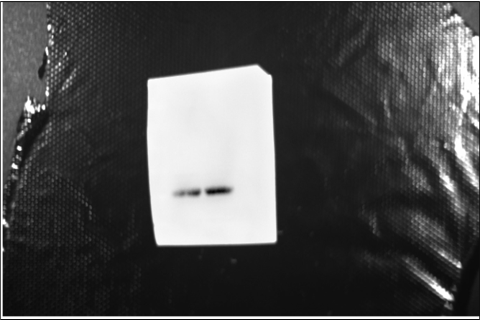

Supplement: Figure 6—source data 7. [file elife-104772-fig6-data7.zip › Figure 6-source data 7/Figure 6E-3.tif]

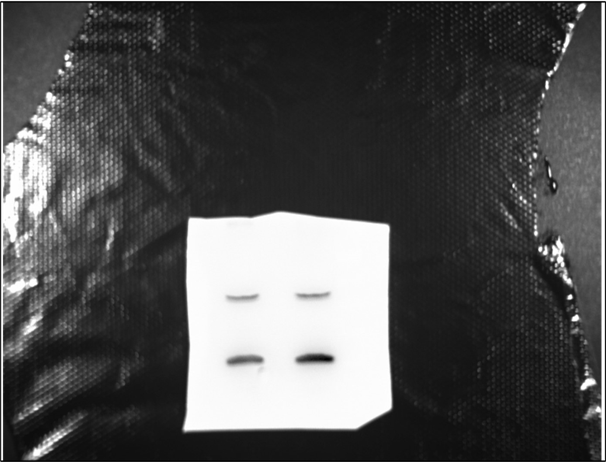

Supplement: Figure 6—source data 7. [file elife-104772-fig6-data7.zip › Figure 6-source data 7/Figure 6E-4.tif]

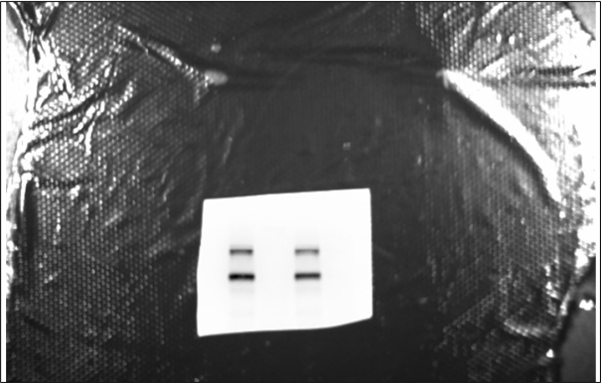

Supplement: Figure 6—source data 7. [file elife-104772-fig6-data7.zip › Figure 6-source data 7/Figure 6E-5.tif]

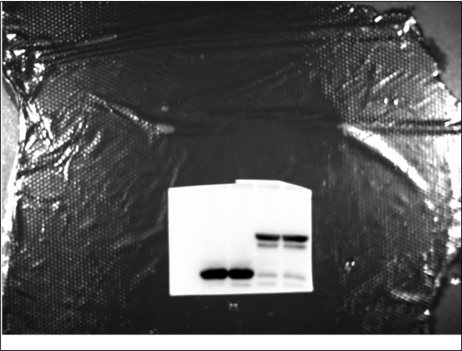

Supplement: Figure 6—source data 7. [file elife-104772-fig6-data7.zip › Figure 6-source data 7/Figure 6E-6.tif]

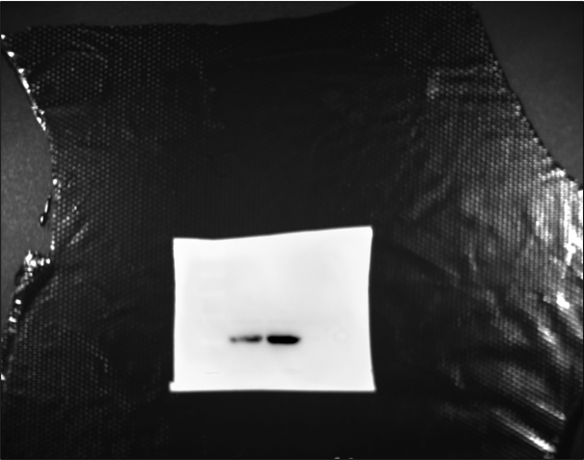

Supplement: Figure 6—source data 7. [file elife-104772-fig6-data7.zip › Figure 6-source data 7/Figure 6E-7.tif]

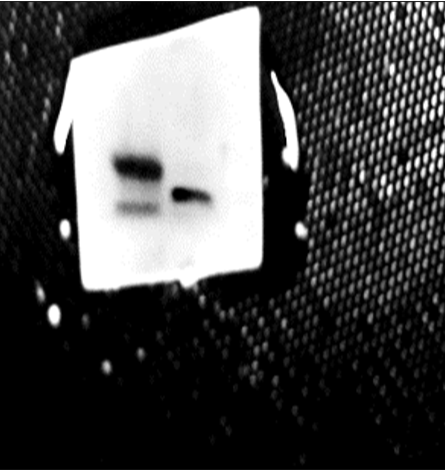

Supplement: Figure 7—source data 1. [file elife-104772-fig7-data1.zip › Figure 7-source data 1/Figure 7A-1.tif]

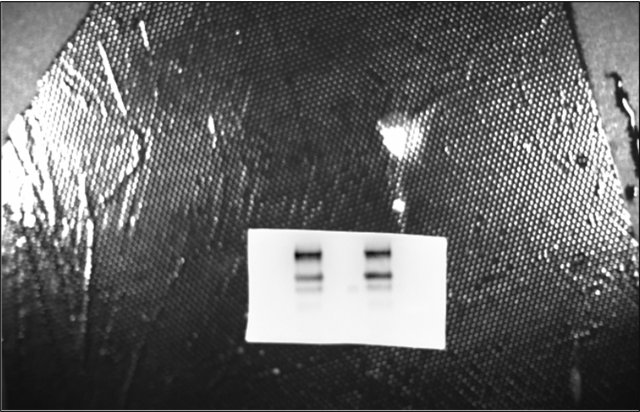

Supplement: Figure 7—source data 1. [file elife-104772-fig7-data1.zip › Figure 7-source data 1/Figure 7A-2.tif]

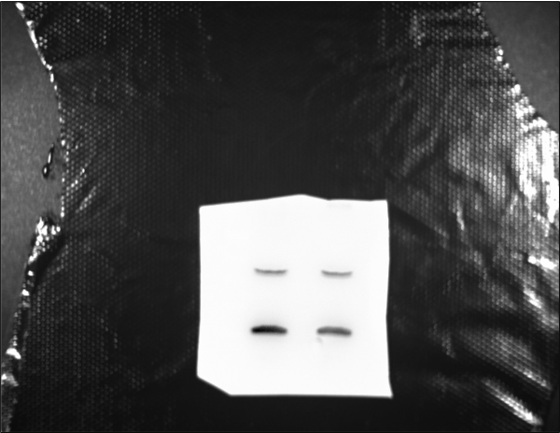

Supplement: Figure 7—source data 1. [file elife-104772-fig7-data1.zip › Figure 7-source data 1/Figure 7A-3.tif]

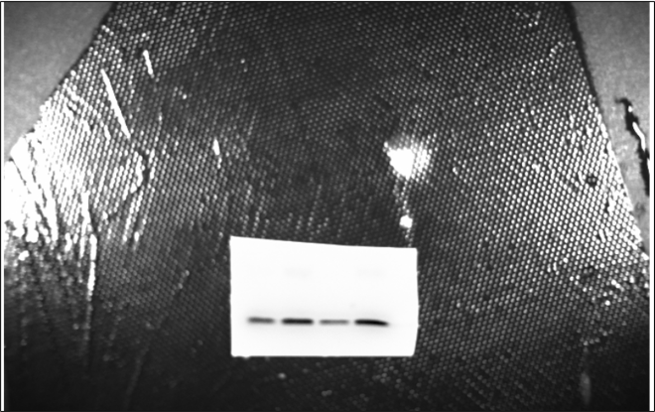

Supplement: Figure 7—source data 1. [file elife-104772-fig7-data1.zip › Figure 7-source data 1/Figure 7A-4.tif]

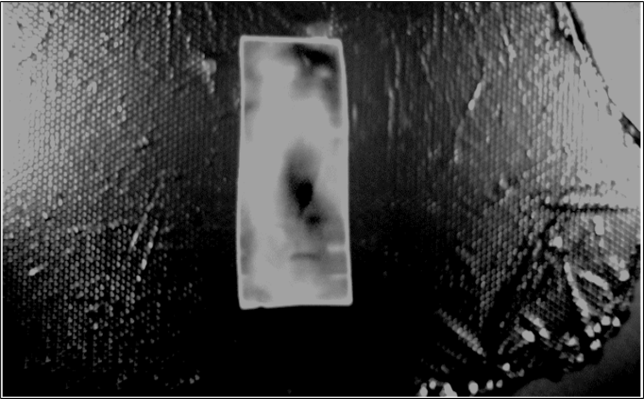

Supplement: Figure 7—source data 3. [file elife-104772-fig7-data3.zip › Figure 7-source data 3/Figure 7B-1.tif]

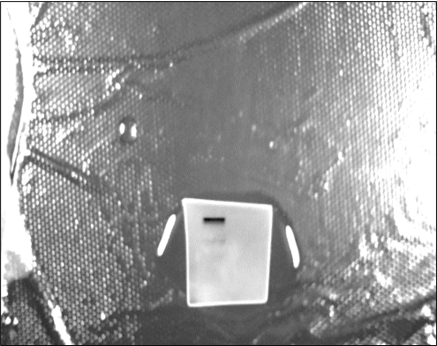

Supplement: Figure 7—source data 3. [file elife-104772-fig7-data3.zip › Figure 7-source data 3/Figure 7B-2.tif]

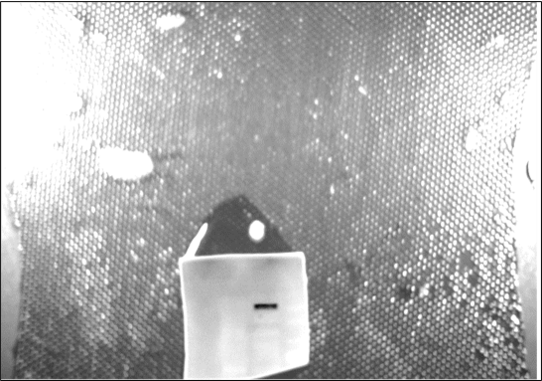

Supplement: Figure 7—source data 3. [file elife-104772-fig7-data3.zip › Figure 7-source data 3/Figure 7B-3.tif]

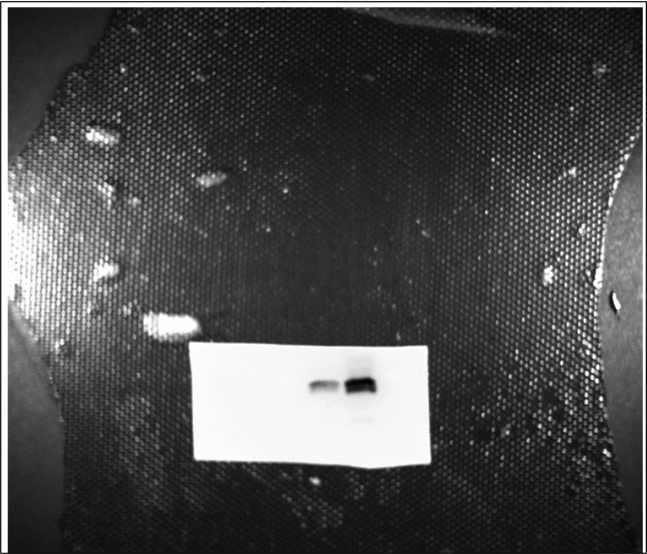

Supplement: Figure 7—source data 3. [file elife-104772-fig7-data3.zip › Figure 7-source data 3/Figure 7B-4.tif]

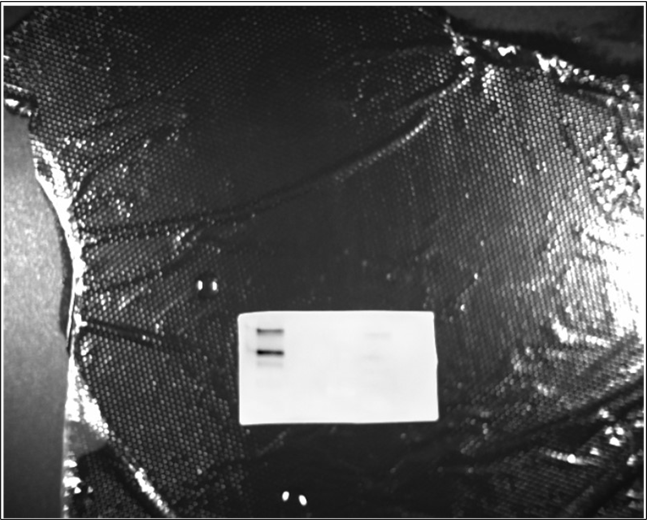

Supplement: Figure 7—source data 3. [file elife-104772-fig7-data3.zip › Figure 7-source data 3/Figure 7B-5.tif]

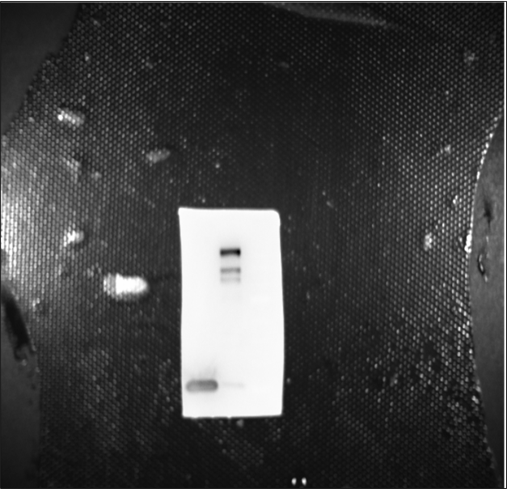

Supplement: Figure 7—source data 3. [file elife-104772-fig7-data3.zip › Figure 7-source data 3/Figure 7B-6.tif]

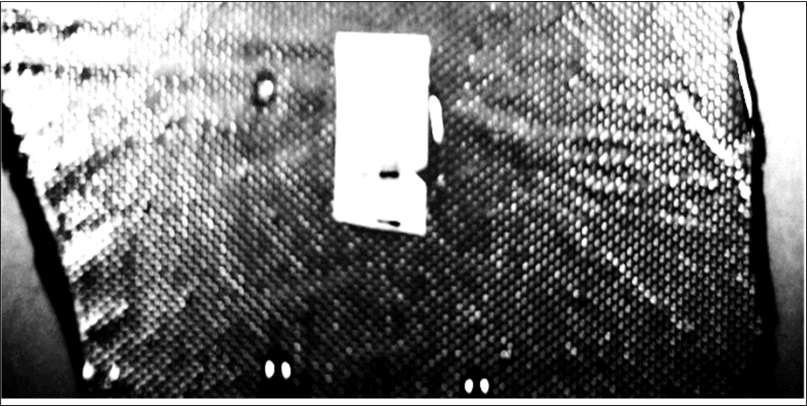

Supplement: Figure 7—source data 5. [file elife-104772-fig7-data5.zip › Figure 7-source data 5/Figure 7C-1.tif]

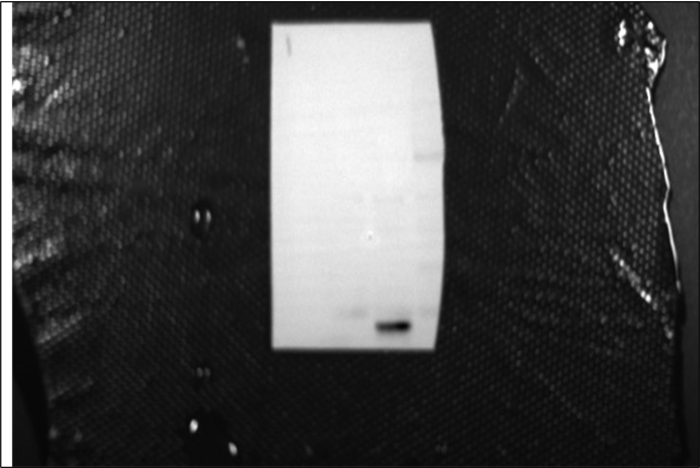

Supplement: Figure 7—source data 5. [file elife-104772-fig7-data5.zip › Figure 7-source data 5/Figure 7C-2.tif]

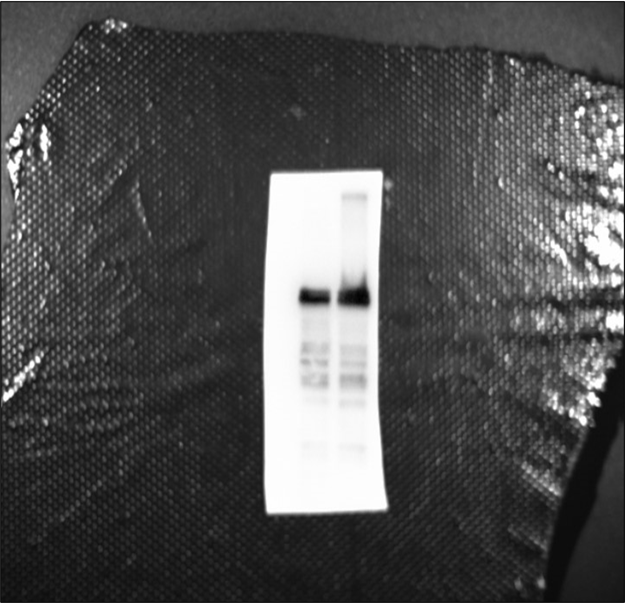

Supplement: Figure 7—source data 5. [file elife-104772-fig7-data5.zip › Figure 7-source data 5/Figure 7C-3.tif]

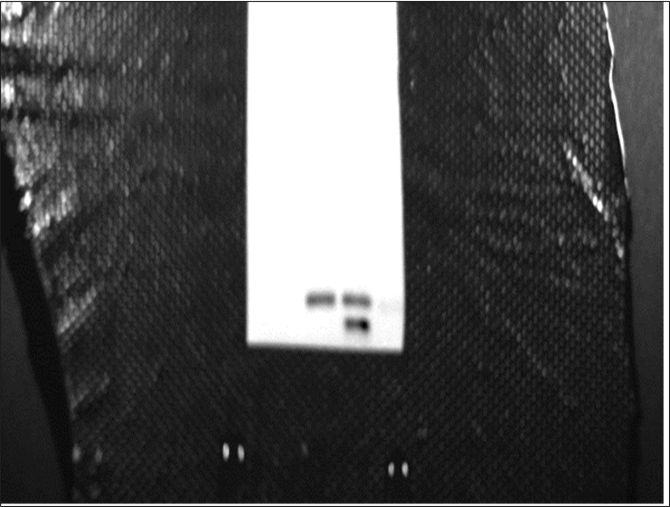

Supplement: Figure 7—source data 5. [file elife-104772-fig7-data5.zip › Figure 7-source data 5/Figure 7C-4.tif]

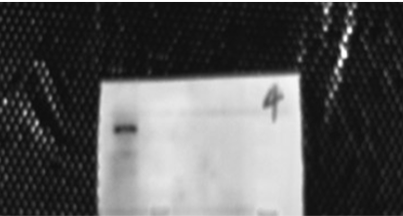

Supplement: Figure 7—source data 7. [file elife-104772-fig7-data7.zip › Figure 7-source data 7/Figure 7D-1.tif]

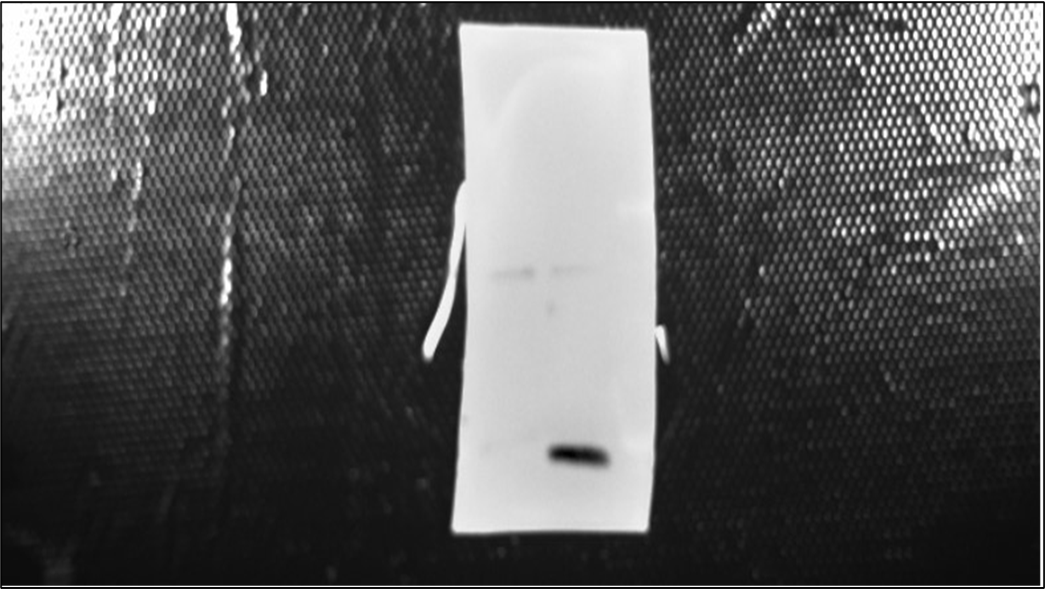

Supplement: Figure 7—source data 7. [file elife-104772-fig7-data7.zip › Figure 7-source data 7/Figure 7D-2.tif]

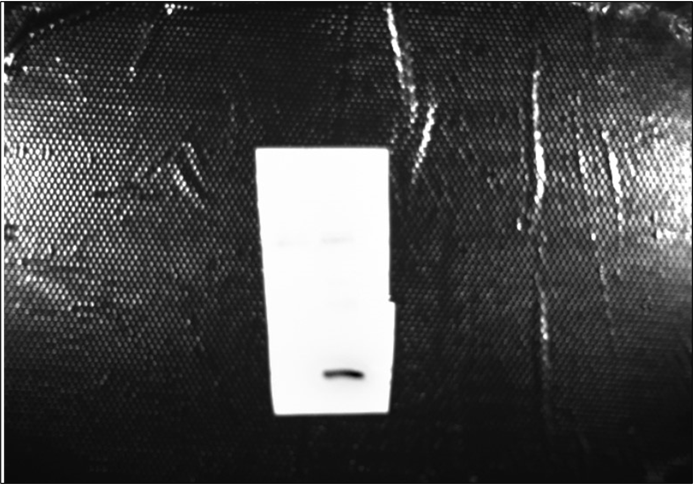

Supplement: Figure 7—source data 7. [file elife-104772-fig7-data7.zip › Figure 7-source data 7/Figure 7D-3.tif]
