# Supplementary material for: Myosin light chain 3 serves as a receptor for nervous necrosis virus entry into host cells via the macropinocytosis pathway
Source: eLife. 2025 Jun 25;13:RP104772. doi: 10.7554/eLife.104772 (PMC12194134; doi:10.7554/eLife.104772)
Supplement: Figure 1—source data 10. [file elife-104772-fig1-data10.pdf]

F

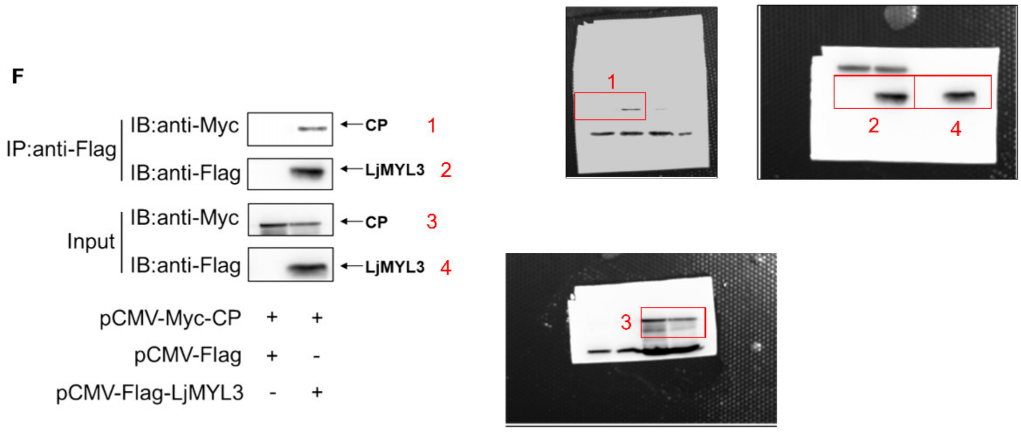

Figure 1, Source Data 4. Original membranes corresponding to Figure 1F. The red box marked as 1 represents the CP of the IP group. The red box marked as 2 represents the LjMYL3 of the IP group. The red box marked as 3 represents the CP of the input group. The red box marked as 4 represents the LjMYL3 of the input group.
