# Supplementary material for: Myosin light chain 3 serves as a receptor for nervous necrosis virus entry into host cells via the macropinocytosis pathway
Source: eLife. 2025 Jun 25;13:RP104772. doi: 10.7554/eLife.104772 (PMC12194134; doi:10.7554/eLife.104772)
Supplement: Figure 2—source data 2. [file elife-104772-fig2-data2.pdf]

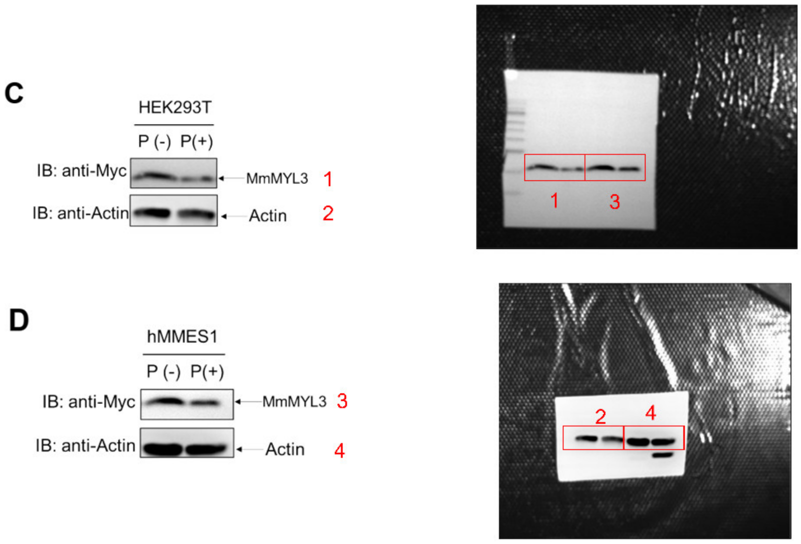

Figure 2, Source Data 1. Original membranes corresponding to Figure 2C and D. The red box marked as 1 represents the MmMYL3 of HEK293T cells. The red box marked as 2 represents the actin of HEK293T cells. The red box marked as 3 represents the MmMYL3 of hMMES1 cells. The red box marked as 4 represents the MmMYL3 of hMMES1 cells.
