# Supplementary material for: Myosin light chain 3 serves as a receptor for nervous necrosis virus entry into host cells via the macropinocytosis pathway
Source: eLife. 2025 Jun 25;13:RP104772. doi: 10.7554/eLife.104772 (PMC12194134; doi:10.7554/eLife.104772)
Supplement: Figure 3—figure supplement 1—source data 2. [file elife-104772-fig3-figsupp1-data2.pdf]

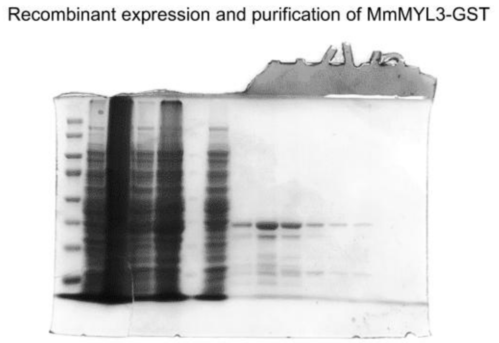

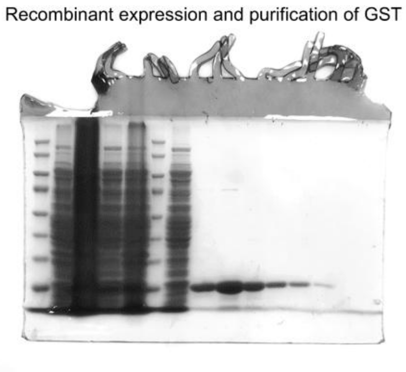

Figure 3-supplement 1, Source Data 1. Original gels corresponding to Figure 3 supplement 1B. Recombinant expression and purification of MmMYL3-GST (left) and GST (right). The sequence of the bands is as follows: marker; cell extracts before IPTG induction; cell extracts after IPTG induction; supernatant after centrifugation; precipitate after centrifugation; flow through; purified recombinant.
