# Supplementary material for: Myosin light chain 3 serves as a receptor for nervous necrosis virus entry into host cells via the macropinocytosis pathway
Source: eLife. 2025 Jun 25;13:RP104772. doi: 10.7554/eLife.104772 (PMC12194134; doi:10.7554/eLife.104772)
Supplement: Figure 3—figure supplement 1—source data 4. [file elife-104772-fig3-figsupp1-data4.pdf]

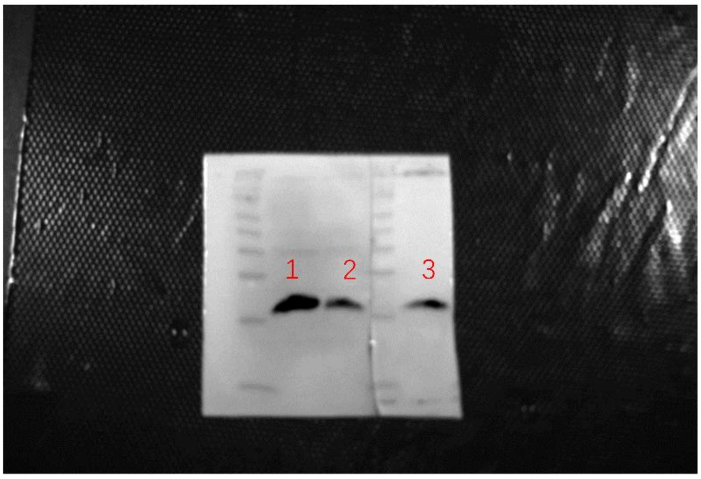

Figure 3-supplement 1, Source Data 2. Original membranes corresponding to Figure 3 supplement 1C. MYL3 antibody validation: 1, HEK 293T cell lysate; 2, hMMES1 cell lysate; 3, MmMYL3 overexpressing hMMES1 cell lysate.
