# Supplementary material for: Myosin light chain 3 serves as a receptor for nervous necrosis virus entry into host cells via the macropinocytosis pathway
Source: eLife. 2025 Jun 25;13:RP104772. doi: 10.7554/eLife.104772 (PMC12194134; doi:10.7554/eLife.104772)
Supplement: Figure 4—source data 2. [file elife-104772-fig4-data2.pdf]

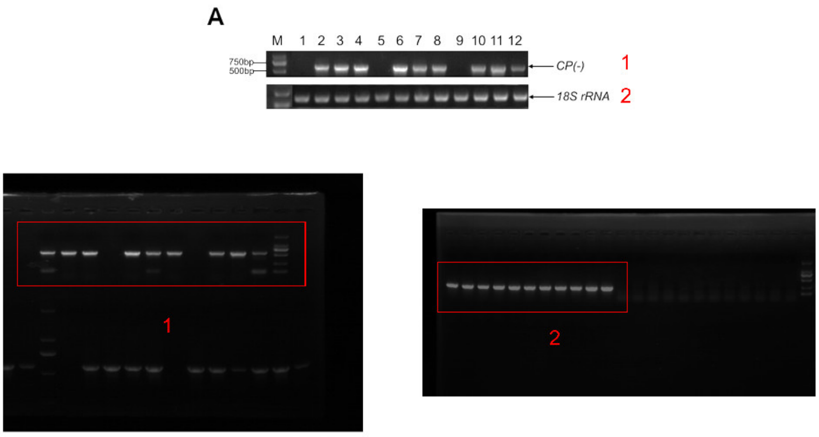

Figure 4, Source Data 1. Original gels corresponding to Figure 4A. The red box marked as 1 represents the CP (-) sequence. The red box marked as 2 represents 18S RNA sequence.
