# Supplementary material for: Myosin light chain 3 serves as a receptor for nervous necrosis virus entry into host cells via the macropinocytosis pathway
Source: eLife. 2025 Jun 25;13:RP104772. doi: 10.7554/eLife.104772 (PMC12194134; doi:10.7554/eLife.104772)
Supplement: Figure 6—source data 6. [file elife-104772-fig6-data6.pdf]

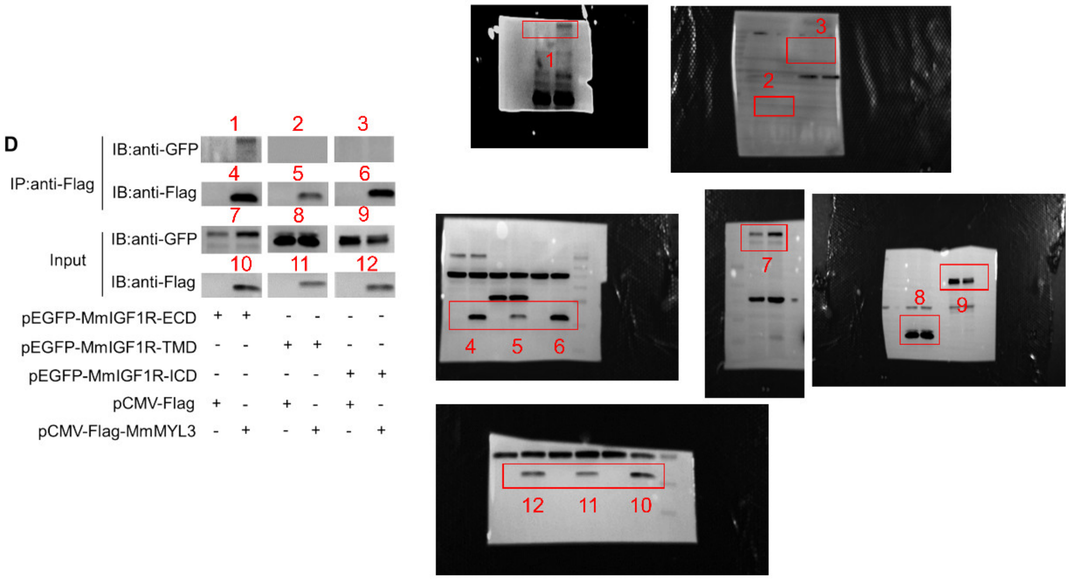

Figure 6, Source Data 2. Original membranes corresponding to Figure 6D. The red box marked as 1 represents the MmIGF1R-ECD of the IP group. The red box marked as 2 represents the MmIGF1R-TMD of the IP group. The red box marked as 3 represents the MmIGF1R-ICD of the IP group. The red box marked as 4, 5 and 6 represents the MmMYL3 of the IP group. The red box marked as 7 represents the MmIGF1R-ECD of the input group. The red box marked as 8 represents the MmIGF1R-TMD of the input group. The red box marked as 9 represents the MmIGF1R-ICD of the input group. The red box marked as 10, 11 and 12 represents the MmMYL3 of the input group.
