# Supplementary material for: Myosin light chain 3 serves as a receptor for nervous necrosis virus entry into host cells via the macropinocytosis pathway
Source: eLife. 2025 Jun 25;13:RP104772. doi: 10.7554/eLife.104772 (PMC12194134; doi:10.7554/eLife.104772)
Supplement: Figure 7—source data 2. [file elife-104772-fig7-data2.pdf]

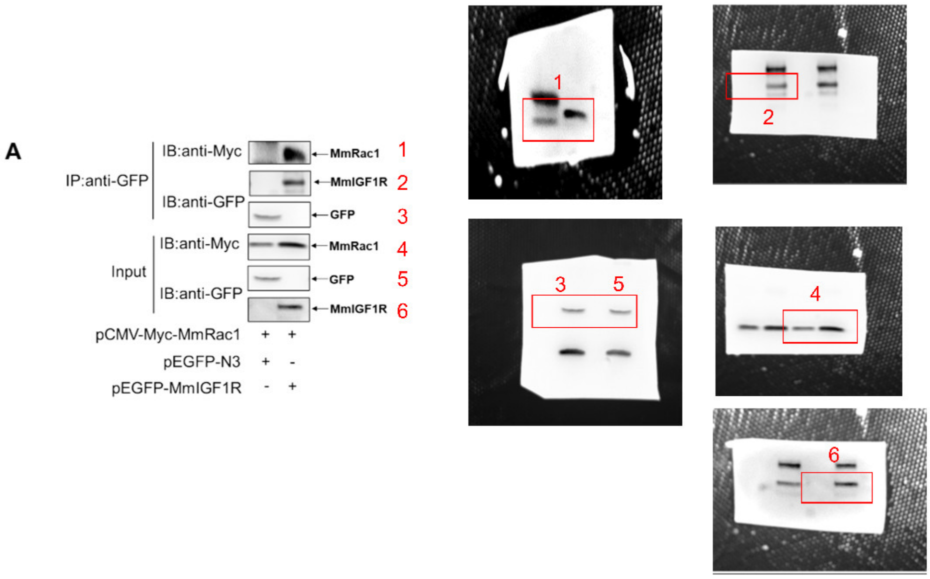

Figure 7, Source Data 1. Original membranes corresponding to Figure 7A. The red box marked as 1 represents the MmRac1 of the IP group. The red box marked as 2 represents the MmIGF1R of the IP group. The red box marked as 3 represents the GFP of the IP group. The red box marked as 4 represents the MmRac1 of the input group. The red box marked as 5 represents the GFP of the input group. The red box marked as 6 represents the MmIGF1R of the input group.
